# The Validation of a Novel, Sex-Specific LDL-Cholesterol Equation and the Friedewald, Sampson-NIH, and Extended-Martin–Hopkins Equations Against Direct Measurement in Korean Adults

**DOI:** 10.3390/metabo15010018

**Published:** 2025-01-05

**Authors:** Hyun Suk Yang, Soo-Nyung Kim, Seungho Lee, Mina Hur

**Affiliations:** 1 Department of Cardiovascular Medicine, Research Institute of Medical Science, Konkuk University School of Medicine, Seoul 05029, Republic of Korea; yang.hyun@kuh.ac.kr; 2 Department of Obstetrics and Gynecology, Konkuk University School of Medicine, Seoul 05029, Republic of Korea; dr.snkim@gmail.com; 3 Department of Preventive Medicine, College of Medicine, Dong-A University, Busan 49201, Republic of Korea; lgydr1@gmail.com; 4 Department of Laboratory Medicine, Konkuk University School of Medicine, Seoul 05030, Republic of Korea

**Keywords:** low-density lipoprotein cholesterol, equations, sex specific, Friedewald, Sampson-NIH, Martin–Hopkins

## Abstract

**Background/Objectives**: The currently established equations for calculating low-density lipoprotein cholesterol (LDLc) do not reflect the sex-specific differences in lipid metabolism. We aimed to develop a sex-specific LDLc equation (SSLE) and validate it with three established equations (Friedewald, Sampson-NIH, and ext-Martin–Hopkins) against direct LDLc measurement in Korean adults. **Methods:** This study included 23,757 subjects (51% male; median age, 51 years) from the 2009–2022 Korean National Health and Nutrition Examination Survey. We developed the SSLE through multiple linear regression incorporating total cholesterol (TC), high-density lipoprotein cholesterol (HDLc), triglycerides (TG), and sex. The validation metrics included Bland–Altman analysis for mean absolute percentage error (MAPE) and agreement of the categorization based on the NCEP ATP-III guidelines, assessed by sex and lipid subgroups. **Results:** The derived SSLE equation was as follows: for TG < 200 mg/dL, LDLc = 0.963 × TC − 0.881 × HDLc − 0.111 × TG + 0.982 × Sex − 6.958; for TG ≥ 200 mg/dL, LDLc = 0.884 × TC − 0.646 × HDLc − 0.126 × TG + 3.742 × Sex − 3.214 (male = 1, female = 0). The MAPE was similar between males and females for the SSLE (4.6% for both) and ext-Martin–Hopkins (5.0% vs. 4.9%) but higher in males for the Sampson-NIH (5.4% vs. 4.9%) and Friedewald (7.6% vs. 5.7%). In the TG ≥ 400 mg/dL group, the MAPE increased progressively: SSLE (10.2%), ext-Martin–Hopkins (12.0%), Sampson-NIH (12.7%), and Friedewald (27.4%). In the LDLc < 70 mg/dL group, the MAPE was as follows: SSLE (8.0%), Sampson-NIH (8.6%), ext-Martin–Hopkins (9.7%), and Friedewald (12.8%). At TG 200–400 mg/dL, the SSLE revealed very good agreement (κ = 0.801) versus good agreement for other equations (ext-Martin–Hopkins κ = 0.794, Sampson-NIH κ = 0.782, Friedewald κ = 0.696). **Conclusions:** The novel SSLE demonstrated superior accuracy and agreement in Korean adults. Further validation studies across different ethnic populations are warranted.

## 1. Introduction

The critical role of low-density lipoprotein cholesterol (LDLc) in cardiovascular health has made its accurate measurement a cornerstone of both primary and secondary prevention strategies for atherosclerotic cardiovascular diseases [1,2]. Direct measurement and indirect calculation are two fundamental approaches to determining LDLc levels [3]. Direct measuring techniques, like ultracentrifugation and homogeneous assays, provide accurate results, but the additional costs and their time-consuming nature limit their application [4,5]. Indirect calculations are often used in clinical practice, estimating LDLc levels using equations incorporating other lipid parameters such as total cholesterol (TC), high-density lipoprotein cholesterol (HDLc), and triglycerides (TG). Discrepancies between calculated and directly measured LDLc levels are often observed, particularly under certain conditions. This can lead to the misclassification of cardiovascular risk and inappropriate treatment decisions.

The Friedewald equation [6], introduced in 1972, has been the most widely used equation for estimating LDLc levels. It is simple and easy to apply, but its accuracy is questionable when TG levels exceed 400 mg/dL or LDLc levels fall below 70 mg/dL [7]. Recent equations have improved the accuracy of LDLc estimation, but they are more complex than the Friedewald equation. The Sampson-NIH equation published in 2020 [8] applies to TG levels up to 800 mg/dL and low LDLc levels within the range we see in clinics. The ext-Martin–Hopkins equation published in 2021 [9], which extends the Martin–Hopkins equation of 2013 [7], offered greater accuracy at TG levels of 400 to 799 mg/dL and less underestimation at low LDLc levels than Friedewald and Sampson-NIH equations [9].

The best choice for LDLc calculation remains debated. One report advocates the Sampson-NIH equation as the preferred method [10], while another supports the ext-Martin–Hopkins equation [11].

Most clinical laboratories in Korea still use the Friedewald equation and seldom adopt newer equations for specific conditions. Furthermore, the validation of these equations in Korean populations remains incomplete—the 2021 study [12] did not include the two most recently recommended equations (Sampson-NIH, ext-Martin–Hopkins). In addition, the potential impacts of ethnic and sex differences require consideration [13,14,15,16], as existing equations do not account for sex-specific variations in lipid metabolism.

We aimed to develop a sex-specific LDLc equation (SSLE) and validate it with three established equations (Friedewald, Sampson-NIH, and ext-Martin–Hopkins) against direct LDLc measurements in Korean adults.

## 2. Materials and Methods

### 2.1. Study Design and Subject Selection

This retrospective cohort study analyzed the de-identified data from the Korean National Health and Nutrition Examination Survey (KNHANES) conducted between 2009 and 2022. We included subjects with valid lipid results, specifically those with direct LDLc measurements obtained using the enzymatic method (Labospect 008AS, Hitachi, Tokyo, Japan). We excluded subjects with TG levels ≥ 800 mg/dL or those under 19 years of age. The final analysis comprised 23,757 subjects.

**Figure 1:**
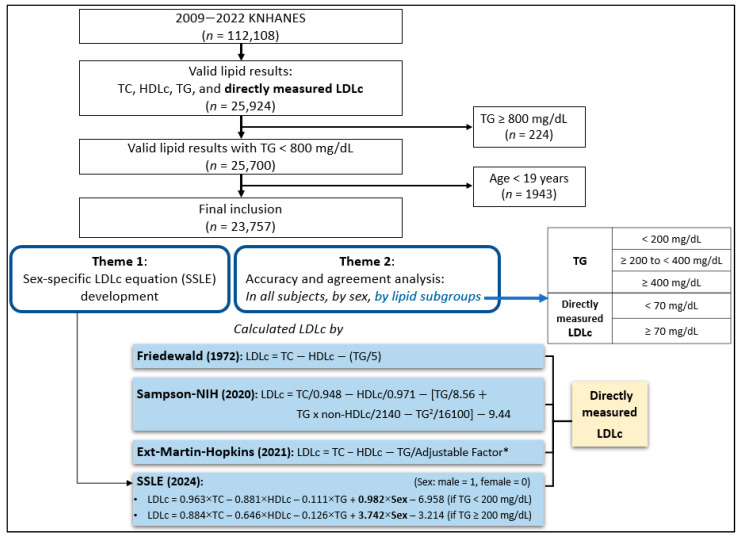
Study flow diagram: * 414 cells (174 cells with TG levels ≤ 399 mg/dL using the 2013 Martin–Hopkins equation; 240 cells with TG levels 400–799 mg/dL using the 2021 ext-Martin–Hopkins equation). Conversion factors: TG (×0.01129 for mmol/L), LDLc (×0.02586 for mmol/L). Abbreviations: KNHANES, Korean National Health and Nutrition Examination Survey; TC, total cholesterol; HDLc, high-density lipoprotein cholesterol; TG, triglycerides; LDLc, low-density lipoprotein cholesterol.

This study had two primary objectives: (1) to develop a novel SSLE equation using Korean population data and (2) to evaluate the accuracy and agreement between SSLE and three established LDLc equations (Friedewald, Sampson-NIH, and ext-Martin–Hopkins) compared to directly measured LDLc. The analysis was performed on the total population, by sex, and across lipid subgroups. For lipid subgroup analysis, subjects were categorized by TG levels (<200 mg/dL, ≥200 to <400 mg/dL, and ≥400 to <800 mg/dL) and LDLc levels (<70 mg/dL and ≥70 mg/dL). The study flow diagram is presented in Figure 1.

### 2.2. Statistical Analysis

Data are expressed as frequency (percentage) for categorical variables and median with interquartile range for continuous variables. For comparison between males and females, we used the chi-square test for categorical variables and the Mann–Whitney U test for continuous variables.

To develop the SSLE, we used multiple linear regression analysis. The dependent variable was measured-LDLc; the independent variables were TC, HDLc, and TG, with or without a sex variable (male = 1, female = 0). The linearity and multicollinearity were checked. As linearity was insufficient between LDLc and HDL (r = 0.007, *p* = 0.32) or TG (r = 0.013, *p* = 0.04), we conducted multiple linear regression analyses separately for two groups (TG < 200 mg/dL, TG ≥ 200 mg/dL).

We calculated the LDLc levels using the four equations in Figure 1. In each equation, the difference between the calculated and the measured LDLc levels was compared by sex using the Mann–Whitney U test. A bivariate correlation analysis was performed with Pearson correlation coefficients between calculated and measured LDLc. The absolute magnitude of the correlation coefficient is interpreted as below: negligible (0.00–0.10), weak (0.10–0.39), moderate (0.40–0.69), strong (0.70–0.89), and very strong (0.90–1.00) correlation [17].

Accuracy and agreement were determined using directly measured LDLc as the reference value. Using the comparison of multiple methods, an extension of the Bland–Altman plot, we obtained the systemic differences [mean, 95% confidence interval (CI)] and absolute percentage error (median, 95% CI). If the CIs do not overlap between the two, they are considered statistically significantly different. The systemic differences and absolute percentage error were compared in total, by sex, and by lipid subgroups. For inter-rater agreement (kappa) analysis, the categorization was modified from the National Cholesterol Education Program Adult Treatment Panel III (NCEP ATP-III) guidelines [18] as six categories of LDLc levels (<70 mg/dL; ≥70 to <100 mg/dL; ≥100 to <130 mg/dL; ≥130 to <160 mg/dL; ≥160 to <190 mg/dL; ≥190 mg/dL). The linear weighted kappa values are interpreted as below: poor (≤0.200), fair (0.201–0.400), moderate (0.401–0.600), good (0.601–0.800), and very good (0.801–1.000) strength of agreement [19].

We used MedCalc^®^ Statistical Software version 23.0.2 (MedCalc Software Ltd., Ostend, Belgium; https://www.medcalc.org; accessed on 5 January 2024). Values of *p* (two-tailed) less than 0.05 were considered statistically significant.

## 3. Results

### 3.1. Subject Characteristics

Of the 23,757 subjects, 12,094 (50.9%) were male and 11,663 (49.1%) were female. The median age was 51 years. Analysis of the lipid profiles revealed significant sex-based differences: HDLc levels were significantly higher in females than in males (51 [43; 62] mg/dL vs. 44 [37; 52] mg/dL, *p* < 0.001), while TG levels showed the opposite pattern, being significantly higher in males than in females (202 [108; 268] mg/dL vs. 118 [75; 214] mg/dL, *p* < 0.001). The detailed subject characteristics stratified by sex are presented in Table 1.

### 3.2. Development of a Sex-Specific LDLc Equation

The detailed results from the multiple linear regression analyses, both with and without sex as a variable, can be found in Supplementary Material Figure S1. The sex-specific model demonstrated an improved predictive power, with adjusted R^2^ values of 0.9568 for subjects with TG < 200 mg/dL and 0.9003 for subjects with TG ≥ 200 mg/dL. These values represent increases of adjusted R^2^ values by 0.02% and 0.25%, respectively, compared to the model without sex differentiation. The resulting SSLE was derived as follows (male = 1, female = 0):

For TG < 200 mg/dL, LDLc = 0.963 × TC − 0.881 × HDLc − 0.111 × TG + 0.982 × Sex − 6.958.

For TG ≥ 200 mg/dL, LDLc = 0.884 × TC − 0.646 × HDLc − 0.126 × TG + 3.742 × Sex − 3.214.

### 3.3. Comparison of Calculated Versus Measured LDLc Levels by Sex

The analysis of the LDLc levels calculated using the four equations is summarized in Table 2. The direct measurement revealed no significant sex-based differences in LDLc levels (*p* = 0.231). However, the Friedewald and Sampson-NIH equations systematically underestimated the LDLc levels in males compared to females (*p* < 0.001). When evaluating the discrepancy between the calculated and the measured LDLc levels, all three established equations exhibited a significant negative bias for males (*p* < 0.05). The newly developed SSLE, however, demonstrated no sex-based bias (*p* = 0.89).

### 3.4. Accuracy Analysis of the LDLc Equations

**Figure 2:**
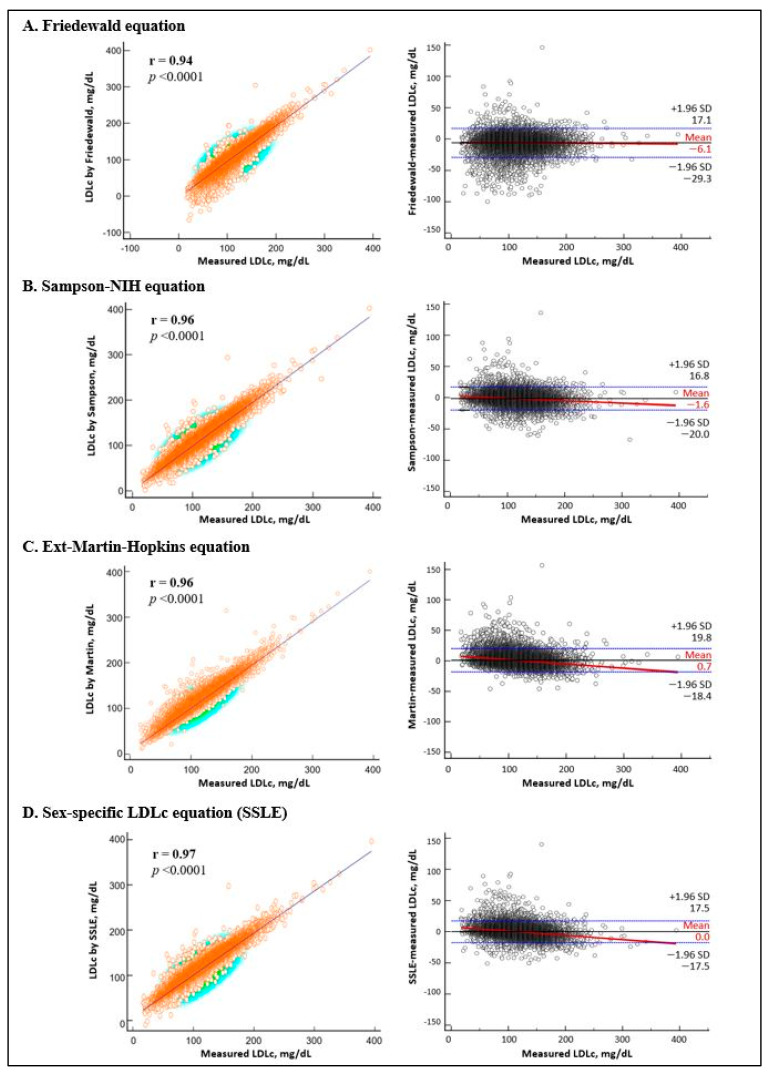
Relationship between four LDLc equations and direct LDLc measurement in all subjects. Left column: Pearson correlation coefficients (r) between calculated and measured LDLc; right column: Bland–Altman plots for the LDLc equations and direct LDLc measurement (reference). To convert LDLc mg/dL to mmol/L, multiply by 0.02586. LDLc, low-density lipoprotein cholesterol; SD, standard deviation.

All four LDLc estimation equations showed very strong correlations with directly measured LDLc levels (Pearson r > 0.9; Figure 2). However, analysis of mean systematic differences revealed varying degrees of bias across equations: Friedewald (−6.11 mg/dL; 95% CI: −6.26 to −5.96), Sampson-NIH (−1.61 mg/dL; −1.72 to −1.49), ext-Martin-Hopkins (0.67 mg/dL; 0.54 to 0.79), and SSLE (0.005 mg/dL; −0.11 to 0.12). Notably, only the SSLE equation demonstrated no significant systematic bias, as its 95% CI crossed zero. The MAPE values for Friedewald, Sampson-NIH, ext-Martin-Hopkins, and SSLE equations were 6.58%, 5.14%, 4.90%, and 4.59%, respectively. Detailed analyses of mean systematic differences and MAPE stratified by sex and lipid subgroups are presented in Table 3 and Table 4.

The sex-stratified analysis revealed that males had a significantly higher MAPE than females for both the Friedewald (7.64% vs. 5.71%) and the Sampson-NIH equations (5.44% vs. 4.86%), with corresponding underestimation biases of −8.61 mg/dL vs. −3.52 mg/dL and −3.06 mg/dL vs. −0.10 mg/dL, respectively. In contrast, no significant sex-based differences in MAPE were observed for the ext-Martin–Hopkins (4.95% vs. 4.85%) or the SSLE equations (4.60% vs. 4.59%). The Bland–Altman plots stratified by sex are provided in Supplementary Material Figure S2.

An analysis stratified by the TG levels revealed that MAPE increased progressively with TG concentrations across all four equations (Supplementary Material Figure S3 for Bland–Altman plots). In the TG < 200 mg/dL group, all the equations maintained MAPE values below 5%, with the SSLE showing the lowest value (4.15%), followed by statistically comparable values for Sampson-NIH (4.44%) and ext-Martin–Hopkins (4.48%), and the highest values for the Friedewald equation (4.95%). In the TG 200–400 mg/dL group, the MAPE values were as follows: SSLE (5.11%), ext-Martin–Hopkins (5.28%), Sampson-NIH (6.09%), and Friedewald (9.68%). While the SSLE and ext-Martin–Hopkins showed comparable MAPE values, they differed in systematic bias (−0.02 mg/dL vs. 3.19 mg/dL, respectively). In the TG > 400 mg/dL group, the SSLE demonstrated the lowest MAPE (10.16%), followed by ext-Martin–Hopkins (11.97%) and Sampson-NIH (12.70%); the latter two showed comparable MAPE values but opposite systematic biases (+10.24 mg/dL vs. −7.85 mg/dL, respectively). The Friedewald equation performed the poorest in this group, with the highest MAPE (27.43%) and substantial systematic underestimation (−26.62 mg/dL).

The LDLc-stratified analysis showed that all equations had a higher MAPE in the lower LDLc group (<70 mg/dL), in which the MAPE values from lowest to highest were as follows: SSLE (7.96%), Sampson-NIH (8.56%), ext-Martin–Hopkins (9.68%), and Friedewald (12.81%). Bland–Altman plots stratified by the LDLc subgroups are provided in Supplementary Material Figure S4.

### 3.5. Agreement of Categorization

The analysis of the concordance between the calculated and the directly measured LDLc categories, based on NCEP ATP-III guidelines, is presented in Table 5. Across all subjects, the SSLE (κ = 0.827), ext-Martin–Hopkins (κ = 0.816), and Sampson-NIH (κ = 0.815) equations demonstrated very good agreement, while the Friedewald equation showed good agreement (κ = 0.759).

The sex-specific analysis revealed that, in males, the SSLE (κ = 0.819) and ext-Martin–Hopkins (κ = 0.810) equations maintained very good agreement, whereas the Sampson-NIH (κ = 0.799) and Friedewald (κ = 0.723) equations showed good agreement. In the TG-stratified analysis, all the equations demonstrated very good agreement for subjects with TG < 200 mg/dL. In the TG 200–400 mg/dL group, only the SSLE maintained very good agreement (κ = 0.801), while the ext-Martin–Hopkins (κ = 0.794), Sampson-NIH (κ = 0.782), and Friedewald (κ = 0.696) equations showed good agreement. In subjects with TG ≥ 400 mg/dL, none of the equations achieved very good categorical agreement with the directly measured LDL, with the Friedewald equation showing only moderate agreement (κ = 0.449).

## 4. Discussion

This study represents the first investigation to examine sex as a variable in LDLc estimation equations. Incorporating sex into the SSLE yielded a modest but significant improvement in the adjusted R^2^, showing a 0.25% increase for subjects with TG ≥ 200 mg/dL. The novel aspect of the SSLE lies in its sex-specific adjustment values for males (0.982 mg/dL for TG < 200 mg/dL and 3.742 mg/dL for TG ≥ 200 mg/dL), which successfully address the negative discrepancies observed in males when using other estimation equations (Table 2). The SSLE demonstrated superior accuracy and agreement with the directly measured LDLc compared to the established methods (Friedewald, Sampson-NIH, and ext-Martin–Hopkins equations) across all subject categories, including sex-specific and lipid subgroups analyses.

### 4.1. Why Is the Sex-Specific Equation Necessary?

Since the introduction of the Friedewald equation [6], over 13 indirect LDLc calculation equations have been published [20]. Indirect calculations remain predominant in clinical practice, as evidenced by our KNHANES database 2009–2022, in which only 23% of cases had a direct LDLc measurement. However, no single equation has yet successfully replaced direct measurement. To develop more accurate equations, consideration must extend beyond the traditional lipid parameters to include patient-specific factors such as age, sex, obesity, ethnicity, metabolic conditions, and medications. Among these factors, sex is particularly crucial, given the significant sex-based differences in lipid profiles demonstrated in our study (Table 1). The absence of sex variables in the existing LDLc estimation equations is notably surprising. Sex-specific lipid metabolism is primarily attributed to hormonal influences and evolutionary adaptations [15,16]. Specifically, estrogen contributes to increased HDLc and decreased TG concentrations [15]. Beyond sex hormones, significant sex-based differences exist in fat storage patterns, fatty acid metabolism, and TG handling [16]. Our study successfully incorporated a sex variable into the LDLc equation without encountering multicollinearity issues in multiple linear regression analyses. Our sex-specific LDLc equation (SSLE) represents a departure from traditional approaches such as the Friedewald formula. Instead, the SSLE employs empirically derived coefficients for TC and HDL. These modified coefficients, along with the sex-specific adjustment term, may reflect underlying biological differences in lipid metabolism between males and females. While the SSLE prioritizes predictive accuracy over theoretical interpretability, its superior performance across various scenarios, particularly in high TG (≥200 mg/dL) and low LDL (<70 mg/dL), suggests these mathematical adjustments capture meaningful biological phenomena.

### 4.2. Friedewald Equation: Too Outmoded?

The primary distinction among the various LDLc equations lies in their estimation of very low-density lipoprotein cholesterol (VLDLc). Fixed TG to VLDLc ratios offer simplicity: Friedewald [6] uses 5, Puavilai [21] uses 6, and Vujovic [22] uses 6.85. Our data and another Korean study [12] suggest that a fixed ratio larger than five is more appropriate for Korean populations. The key limitation of fixed-ratio models is their inability to account for the progressive increase in the TG to VLDLc ratio with rising TG levels [10]. Our findings confirm this limitation, demonstrating an increasing MAPE and negative systematic bias with rising TG levels (Table 3 and Table 4). In subjects with TG ≥ 400 mg/dL, we observed a MAPE as high as 27.43% with a systematic bias of −26.62 mg/dL. The poor performance of Friedewald in low LDLc [7] was also confirmed in this Korean population. Based on these findings, we strongly advise against using the Friedewald equation for Korean adults with TG ≥ 400 mg/dL or LDLc < 70 mg/dL due to significant LDL level underestimation.

### 4.3. Sampson-NIH vs. Ext-Martin–Hopkins: Which Is Better in Korean Big Data?

The Martin–Hopkins equation (2013) represented a major advance over the 1972 Friedewald equation by introducing adjustable factors using a 180-cell stratification of TG/non-HDLc ratios for the TG to VLDLc conversion [7]. The ext-Martin–Hopkins equation (2021) expanded this approach to include TG levels of 400–799 mg/dL using 240-cell stratification [9,23], resulting in 414 adjustable factors in our analysis. However, this table-based approach presents implementation challenges and raises concerns about discontinuities, particularly in the inconsistent changes of adjustable factors within the 240-cell table at elevated TG levels. In contrast, the Sampson-NIH equation (2020) offers advantages through its continuous, smooth transitions and simpler laboratory implementation [8].

Our analysis revealed distinct strengths for each equation. The ext-Martin–Hopkins equation showed better sex-specific performance than the Sampson-NIH, particularly in males (MAPE: 4.95% vs. 5.44%; mean systematic bias: 0.61 mg/dL vs. −3.06 mg/dL), possibly explaining its successful validation across diverse populations [7,11,23]. Estimating LDLc in hypertriglyceridemia or low LDLc remains challenging despite recent advancements in calculation methods [24]. At very high TG levels (≥400 mg/dL), both equations showed a similarly high MAPE but opposing directional biases (ext-Martin–Hopkins: +10.24 mg/dL; Sampson-NIH: −7.85 mg/dL). The ext-Martin–Hopkins’ overestimation might stem from its validation against the Vertical Auto Profile method, which tends to underestimate VLDLc in hypertriglyceridemia [9,10]. At low LDLc levels (<70 mg/dL), the Sampson-NIH equation demonstrated superior performance over the ext-Martin–Hopkins equation in both accuracy (MAPE: 8.56% vs. 9.68%) and systematic bias (0.45 mg/dL vs. 5.55 mg/dL). As previously established in the literature [3,7,8,9,10,11], we confirmed that both equations markedly outperformed the traditional Friedewald equation.

### 4.4. Clinical Applications of LDLc Equations

**Figure 3:**
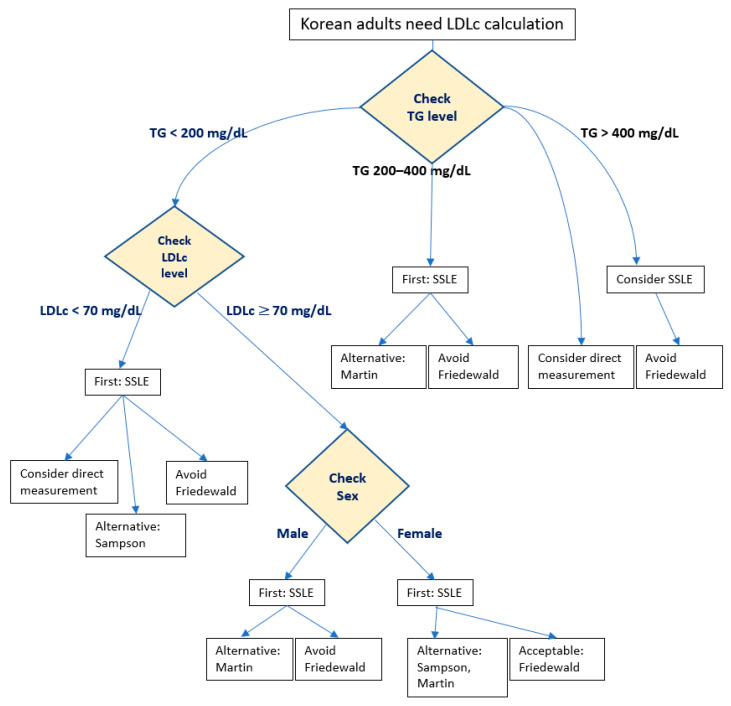
Proposed algorithm to guide the selection of low-density lipoprotein cholesterol (LDLc) equations in Korean adults. Conversion factors: triglycerides (TG) (×0.01129 for mmol/L), LDLc (×0.02586 for mmol/L). SSLE, sex-specific LDLc equation.

Based on our findings (Table 3, Table 4 and Table 5), we propose a clinical algorithm for LDLc estimation in Korean adults (Figure 3). Our analysis suggests that the Friedewald equation should be limited to females with TG < 200 mg/dL and LDLc ≥ 70 mg/dL. For all other scenarios, the SSLE emerges as our primary recommendation, with the ext-Martin–Hopkins equation serving as an alternative for males and the Sampson-NIH equation being suitable for cases with low LDLc. In cases of hypertriglyceridemia, clinicians should note that the ext-Martin–Hopkins equation tends toward overestimation of LDLc, while the Sampson-NIH equation tends toward underestimation.

Several key limitations should be acknowledged. First, our analysis of the KNHANES data had a relatively small sample size, potentially limiting generalizability. Second, while we developed sex-specific equations, the need for separate formulas across TG levels suggests that a more unified approach using advanced statistical methods may be beneficial. Future studies need to explore the biological basis of these coefficients, possibly with measured VLDLc levels, and validate their applicability across different ethnic populations. Third, important confounding factors, including age, hypertension, and BMI, require further examination (Supplementary Material Figure S5), as do specific clinical subgroups such as postmenopausal females, patients with metabolic diseases, and those on lipid-lowering medications.

## 5. Conclusions

The novel sex-specific LDL equation (SSLE) demonstrated superior accuracy and agreement in Korean adults. This study highlights the significance of sex-specific approaches in LDL cholesterol estimation equations, warranting further validation studies across different ethnic populations.

## Figures and Tables

**Table 1:** Subject characteristics: general and lipid profiles by sex.

	Total	Male	Female	*p* Value
Number of subjects, *n* (% of total cohort)	23,757 (100)	12,094 (50.9)	11,663 (49.1)	_
Age, years	51.0 [38.0; 63.0]	50.0 [38.0; 62.0]	53.0 [39.0; 61.0]	<0.001
19–39 years, *n* (%)	6556 (27.6)	3469 (28.7)	3087 (26.5)	<0.001
40–59 years, *n* (%)	9565 (40.3)	5069 (41.9)	4496 (38.5)	
60–79 years, *n* (%)	6972 (29.3)	3296 (27.3)	3676 (31.5)	
≥80 years, *n* (%)	664 (2.8)	260 (2.1)	404 (3.5)	
BMI, kg/m^2^	24.2 [22.0; 26.5]	24.7 [22.7; 26.8]	23.6 [21.3; 26.1]	<0.001
BMI ≥ 25 kg/m^2^, *n* (%)	4033 (17.1)	2417 (20.1)	1.616 (14.0)	<0.001
Smoking, *n* (%)				
Never	12,401 (53.4)	2345 (19.9)	10,056 (88.0)	<0.001
Past	5432 (23.4)	4709 (39.9)	723 (6.3)	
Current	5402 (23.2)	4755 (40.3)	647 (5.7)	
Alcohol, *n* (%)				
None	7577 (36.5)	2608 (23.0)	4969 (52.8)	<0.001
1–4 times/month	7424 (35.8)	4168 (36.8)	3256 (34.6)	
>4 times/month	5742 (27.7)	4560 (40.2)	1182 (12.6)	
Systolic BP, mmHg	119 [109; 131]	121 [112; 132]	116 [105; 130]	<0.001
Diastolic BP, mmHg	76 [70; 83]	79 [72; 86]	73 [67; 80]	<0.001
Hypertension, *n* (%)	9032 (38.0)	5037 (41.6)	3995 (34.3)	<0.001
Diabetes mellitus, *n* (%)	3539 (14.9)	1994 (16.5)	1545 (13.2)	<0.001
**Lipid profiles**				
Total cholesterol, mg/dL	192 [167; 219]	199 [166; 218]	192 [168; 219]	0.005
LDLc, mg/dL	113 [91; 137]	113 [91; 136]	113 [91; 137]	0.231
LDLc < 70 mg/dL, *n* (%)	1957 (8.2%)	1085 (9.0%)	872 (7.5%)	<0.001
HDLc, mg/dL	47 [39; 57]	44 [37; 52]	51 [43; 62]	<0.001
TG, mg/dL	151 [88; 242]	202 [108; 268]	118 [75; 214]	<0.001
TG < 200 mg/dL, *n* (%)	13,945 (58.7)	5912 (48.9)	8033 (68.9)	<0.001
TG ≥ 200 to < 400 mg/dL, *n* (%)	8543 (36.0)	5234 (43.3)	3309 (28.4)	
TG ≥ 400 to < 800 mg/dL, *n* (%)	1269 (5.3)	948 (7.8)	321 (2.8)	

Values are number (%) or median [interquartile range]. Conversion factors: LDLc and HDLc (×0.02586 for mmol/L); TG (×0.01129 for mmol/L). *P* value between males and females: chi-square test or Mann–Whitney U test. Missing data: BMI (*n* = 158, 0.7%), smoking status (*n* = 522, 2.2%), alcohol consumption frequency (*n* = 3014, 12.7%), and BP (*n* = 185, 0.8%). Abbreviations: *n*, number; BMI, body mass index; BP, blood pressure; LDLc, low-density lipoprotein cholesterol; HDLc, high-density lipoprotein cholesterol; TG, triglycerides.

**Table 2:** Four LDLc equations’ calculated levels and their differences from measured levels, stratified by sex.

	Total (*n* = 23,757)	Male (*n* = 12,094)	Female (*n* = 11,663)	*p* Value
**LDLc, mg/dL**				
Measured	113.0 [91.0; 137.0]	113.0 [91.0; 136.0]	113.0 [91.0; 137.0]	0.231
Friedewald	107.2 [84.8; 131.6]	105.0 [82.0; 129.1]	109.7 [87.2;134.0]	<0.001
Sampson-NIH	111.3 [89.5; 135.0]	109.9 [88.3; 133.1]	113.0 [90.6; 137.1]	<0.001
Ext-Martin–Hopkins	113.7 [92.3; 136.4]	113.8 [92.7; 135.7]	113.6 [92.0; 137.4]	0.155
SSLE	113.1 [91.8; 135.8]	113.0 [92.0; 135.2]	113.2 [91.5; 136.5]	0.264
**(Calculated − Measured) LDLc, mg/dL**				
Friedewald − Measured	−5.3 [−11.8; 1.0]	−7.3 [−14.8; −1.0]	−3.4 [−9.1; 2.8]	<0.001
Sampson-NIH − Measured	−1.8 [−7.0; 3.8]	−3.2 [−8.4; 2.4]	−0.5 [−5.7; 4.9]	<0.001
Ext-Martin–Hopkins − Measured	−0.4 [−5.6; 5.4]	−0.7 [−5.7; 5.3]	−0.1 [−5.6; 5.4]	0.044
SSLE − Measured	−0.5 [−5.4; 4.7]	−0.5 [−5.3; 4.8]	−0.5 [−5.4; 4.6]	0.890

Values are median [interquartile range]; *p* values compare males and females using the Mann–Whitney U test. Conversion factor: LDLc (×0.02586 for mmol/L). LDLc, low-density lipoprotein cholesterol; SSLE, sex-specific LDLc equation.

**Table 3:** Accuracy of four LDLc equations compared to direct measurement: systemic differences.

	Total	Sex		Triglycerides			LDLc	
		Male	Female	<200 mg/dL	≥200 to <400 mg/dL	≥400 to <800 mg/dL	<70 mg/dL	≥70 mg/dL
	*n* = 23,757	*n* = 12,094	*n* = 11,663	*n* = 13,945	*n* = 8543	*n* = 1269	*n* = 1957	*n* = 21,800
Friedewald	−6.11 (−6.26; −5.96)	−8.61 (−8.84; −8.38)	−3.52 (−3.70; −3.33)	−2.01 (−2.13; −1.88)	−9.76 (−10.0; −9.52)	−26.62 (−27.8; −25.5)	−6.91 (−7.58; −6.24)	−6.04 (−6.19; −5.89)
Sampson-NIH	−1.61 (−1.72; −1.49)	−3.06 (−3.24; −2.89)	−0.10 (−0.26; 0.07)	−0.18 (−0.30; −0.06)	−3.01 (−3.23; −2.79)	−7.85 (−8.75; −6.94)	0.45 (0.02; 0.87)	−1.79 (−1.92; −1.67)
Ext-Martin–Hopkins	0.67 (0.54; 0.79)	0.61 (0.44; 0.79)	0.72 (0.54; 0.89)	−1.75 (−1.87; −1.64)	3.19 (2.96; 3.42)	10.24 (9.34; 11.15)	5.55 (5.04; 6.06)	0.23 (0.10; 0.35)
SSLE	0.005 (−0.11; 0.12)	0.005 (−0.16; 0.17)	0.005 (−0.15; 0.16)	0.01 (−0.11; 0.12)	−0.02 (−0.24; 0.19)	0.19 (−0.73; 1.11)	3.97 (3.54; 4.41)	−0.35 (−0.47; −0.23)

Values are mean levels (mg/dL) (95% confidence intervals). Conversion factors: triglycerides (×0.01129 for mmol/L), LDLc (×0.02586 for mmol/L). Color coding (mean systemic difference): dark blue (≤−10), light blue (−10 to −2), white (−2 to 2), light red (2 to 10), dark red (≥10). LDLc, low-density lipoprotein cholesterol; SSLE, sex-specific LDLc equation.

**Table 4:** Accuracy of four LDLc equations compared to direct measurement: median absolute percentage error (MAPE).

	Total	Sex		Triglycerides			LDLc	
		Male	Female	<200 mg/dL	≥200 to <400 mg/dL	≥400 to <800 mg/dL	<70 mg/dL	≥70 mg/dL
	*n* = 23,757	*n* = 12,094	*n* = 11,663	*n* = 13,945	*n* = 8543	*n* = 1269	*n* = 1957	*n* = 21,800
Friedewald	6.58% (6.47–6.67)	7.64% (7.47–7.80)	5.71% (5.59–5.84)	4.95% (4.87–5.04)	9.68% (9.45–9.89)	27.43% (26.23–28.46)	12.81% (12.21–13.59)	6.23% (6.14–6.35)
Sampson-NIH	5.14% (5.06–5.22)	5.44% (5.33–5.56)	4.86% (4.76–4.96)	4.44% (4.35–4.53)	6.09% (5.94–6.24)	12.70% (11.83–13.30)	8.56% (8.05–8.94)	4.93% (4.86–5.01)
Ext-Martin–Hopkins	4.90% (4.82–4.98)	4.95% (4.82–5.06)	4.85% (4.77–4.96)	4.48% (4.39–4.57)	5.28% (5.15–5.41)	11.97% (11.33–12.99)	9.68% (9.18–10.32)	4.67% (4.61–4.74)
SSLE	4.59% (4.53–4.67)	4.60% (4.51–4.71)	4.59% (4.50–4.68)	4.15% (4.07–4.22)	5.11% (4.99–5.23)	10.16% (9.58–10.67)	7.96% (7.58–8.45)	4.43% (4.37–4.49)

Values are median (95% confidence intervals). Conversion factors: triglycerides (×0.01129 for mmol/L), LDLc (×0.02586 for mmol/L). Color coding (MAPE): blue (<5%), green (5–6%), yellow (6–8%), orange (8–12%), red (>12%). LDLc, low-density lipoprotein cholesterol; SSLE, sex-specific LDLc equation.

**Table 5:** Agreement of categorization between calculated and directly measured low-density lipoprotein cholesterol.

	Total	Sex		Triglycerides		
		Male	Female	<200 mg/dL	≥200 to <400 mg/dL	≥400 to <800 mg/dL
Friedewald	0.759 (0.754–0.764)	0.723 (0.715–0.730)	0.796 (0.789–0.803)	0.827 (0.821–0.833)	0.696 (0.687–0.705)	0.449 (0.419–0.480)
Sampson-NIH	0.815 (0.810–0.819)	0.799 (0.792–0.805)	0.831 (0.824–0.837)	0.847 (0.842–0.853)	0.782 (0.774–0.790)	0.638 (0.610–0.666)
Ext-Martin–Hopkins	0.816 (0.811–0.820)	0.810 (0.803–0.817)	0.822 (0.815–0.828)	0.843 (0.837–0.848)	0.794 (0.786–0.802)	0.635 (0.606–0.663)
SSLE	0.827 (0.822–0.831)	0.819 (0.813–0.826)	0.834 (0.828–0.840)	0.852 (0.846–0.857)	0.801 (0.793–0.808)	0.696 (0.670–0.722)

Values are linear weighted kappa (95% confidence interval), categorized per the National Cholesterol Education Program Adult Treatment Panel III. Conversion factor: triglycerides (×0.01129 for mmol/L). Strength of agreement: very good (blue), good (green), moderate (yellow), fair (orange), poor (red). SSLE, sex-specific LDLc equation.

## Data Availability

All the data described in this study are available within this article or in its Supplementary Materials.

## Supplementary Materials

The following supporting information can be downloaded at https://www.mdpi.com/article/10.3390/metabo15010018/s1: Figure S1: Multiple linear regression analyses; Figure S2: Bland–Altman plots stratified by sex; Figure S3. Bland–Altman plots stratified by TG subgroups; Figure S4. Bland–Altman plots stratified by LDLc subgroups; Figure S5. Subgroup analysis by age, hypertension, and BMI.
