# Supplementary material for: The Validation of a Novel, Sex-Specific LDL-Cholesterol Equation and the Friedewald, Sampson-NIH, and Extended-Martin–Hopkins Equations Against Direct Measurement in Korean Adults
Source: Metabolites. 2025 Jan 5;15(1):18. doi: 10.3390/metabo15010018 (PMC11767536; doi:10.3390/metabo15010018)
Supplement: Supplementary file 1 [file metabolites-15-00018-s001.zip › Figure S1.pdf]

S1. Multiple linear regression analysis

A. Independent variables: Total cholesterol (TC), HDL cholesterol (HDLc) and Triglycerides (TG)

Dependent Y	LDLd Directly measured LDL cholesterol
Filter	TG less than 200 (TG <200 mg/dL)

Least squares multiple regression

Method	Enter

Sample size	13945
Coefficient of determination R^2	0.9566
R^2-adjusted	0.9566
Multiple correlation coefficient	0.9781
Residual standard deviation	6.8760

Regression Equation

Independent variables	Coefficient	Std. Error	95% CI	t	P	$r_{partial}$	$r_{semipartial}$	VIF
(Constant)	-6.1535	0.3814	-6.9012 to -5.4058	-16.1325	<0.0001			
TC	0.9616	0.001793	0.9581 to 0.9651	536.3279	<0.0001	0.9766	0.9461	1.235
HDL	-0.8879	0.004952	-0.8976 to -0.8782	-179.2923	<0.0001	-0.8352	0.3163	1.346
TG	-0.1095	0.001625	-0.1127 to -0.1063	-67.4068	<0.0001	-0.4958	0.1189	1.331

Analysis of Variance

Source	DF	Sum of Squares	Mean Square
Regression	3	14535366.662	4845122.221
Residual	13941	659126.889	47.280

F-ratio	102477.763
Significance level	P<0.0001

Zero order and simple correlation coefficients

Variable	LDLd	TC	HDL
TC	0.9251		
HDL	-0.04756	0.2561	
TG	0.2441	0.2353	-0.3650

Dependent Y	LDLd Directly measured LDL cholesterol
Filter	TG >=200 (TG ≥200 mg/dL)

Least squares multiple regression

Method	Enter

Sample size	9812
Coefficient of determination R^2	0.8979
R^2-adjusted	0.8978
Multiple correlation coefficient	0.9476
Residual standard deviation	11.4090

Regression Equation

Independent variables	Coefficient	Std. Error	95% CI	t	P	$r_{partial}$	$r_{semipartial}$	VIF
(Constant)	0.05242	0.7768	-1.4703 to 1.5751	0.06748	0.9462			
TC	0.8807	0.003130	0.8745 to 0.8868	281.3269	<0.0001	0.9433	0.9079	1.150
HDL	-0.6652	0.01282	-0.6903 to -0.6401	-51.8748	<0.0001	-0.4640	0.1674	1.178
TG	-0.1243	0.001123	-0.1265 to -0.1221	-110.6673	<0.0001	-0.7452	0.3571	1.077

Analysis of Variance

Source	DF	Sum of Squares	Mean Square
Regression	3	11221400.534	3740466.845
Residual	9808	1276659.578	130.165

F-ratio	28736.321
Significance level	P<0.0001

Zero order and simple correlation coefficients

Variable	LDLd	TC	HDL
TC	0.8737		
HDL	0.1947	0.3134	
TG	-0.2228	0.1159	-0.1925

B. Independent variables: TC, HDLc, TG and Sex (male=1, female=0)

Dependent Y	LDLd	Directly measured LDL cholesterol
Filter	TG less than 200	(TG <200 mg/dL)

Least squares multiple regression

Method	Enter

Sample size	13945
Coefficient of determination R^2	0.9568
R^2-adjusted	0.9568
Multiple correlation coefficient	0.9782
Residual standard deviation	6.8604

Regression Equation

Independent variables	Coefficient	Std. Error	95% CI	t	P	$r_{partial}$	$r_{semipartial}$	VIF
(Constant)	-6.9575	0.3935	-7.7287 to -6.1862	-17.6822	<0.0001			
TC	0.9626	0.001793	0.9591 to 0.9661	536.9039	<0.0001	0.9767	0.9449	1.240
HDL	-0.8814	0.005008	-0.8912 to -0.8716	-175.9887	<0.0001	-0.8304	0.3097	1.383
TG	-0.1109	0.001631	-0.1141 to -0.1077	-68.0338	<0.0001	-0.4993	0.1197	1.347
sex_male	0.9818	0.1221	0.7425 to 1.2210	8.0431	<0.0001	0.06797	0.01416	1.078

Analysis of Variance

Source	DF	Sum of Squares	Mean Square
Regression	4	14538411.348	3634602.837
Residual	13940	656082.204	47.065

F-ratio	77225.633
Significance level	P<0.0001

Zero order and simple correlation coefficients

Variable	LDLd	TC	HDL	TG
TC	0.9251			
HDL	-0.04756	0.2561		
TG	0.2441	0.2353	-0.3650	
sex_male	-0.01397	-0.09003	-0.2466	0.1709

Dependent Y	LDLd	Directly measured LDL cholesterol
Filter	TG >=200	(TG ≥200 mg/dL)

Least squares multiple regression

Method	Enter

Sample size	9812
Coefficient of determination R^2	0.9003
R^2-adjusted	0.9003
Multiple correlation coefficient	0.9489
Residual standard deviation	11.2706

Regression Equation

Independent variables	Coefficient	Std. Error	95% CI	t	P	$r_{partial}$	$r_{semipartial}$	VIF
(Constant)	-3.2141	0.7954	-4.7733 to -1.6549	-4.0406	0.0001			
TC	0.8838	0.003099	0.8778 to 0.8899	285.1912	<0.0001	0.9447	0.9092	1.155
HDL	-0.6463	0.01273	-0.6712 to -0.6213	-50.7824	<0.0001	-0.4563	0.1619	1.189
TG	-0.1261	0.001116	-0.1283 to -0.1240	-113.0568	<0.0001	-0.7522	0.3604	1.090
sex_male	3.7421	0.2399	3.2720 to 4.2123	15.6011	<0.0001	0.1556	0.04974	1.036

Analysis of Variance

Source	DF	Sum of Squares	Mean Square
Regression	4	11252317.884	2813079.471
Residual	9807	1245742.227	127.026

F-ratio	22145.729
Significance level	P<0.0001

Zero order and simple correlation coefficients

Variable	LDLd	TC	HDL	TG
TC	0.8737			
HDL	0.1947	0.3134		
TG	-0.2228	0.1159	-0.1925	
sex_male	-0.05654	-0.08874	-0.1454	0.1226
