# Supplementary material for: The Validation of a Novel, Sex-Specific LDL-Cholesterol Equation and the Friedewald, Sampson-NIH, and Extended-Martin–Hopkins Equations Against Direct Measurement in Korean Adults
Source: Metabolites. 2025 Jan 5;15(1):18. doi: 10.3390/metabo15010018 (PMC11767536; doi:10.3390/metabo15010018)

S3. Bland-Altman Plots Stratified by Triglyceride (TG) Levels

A. TG levels <200 mg/dL (n=13,945)

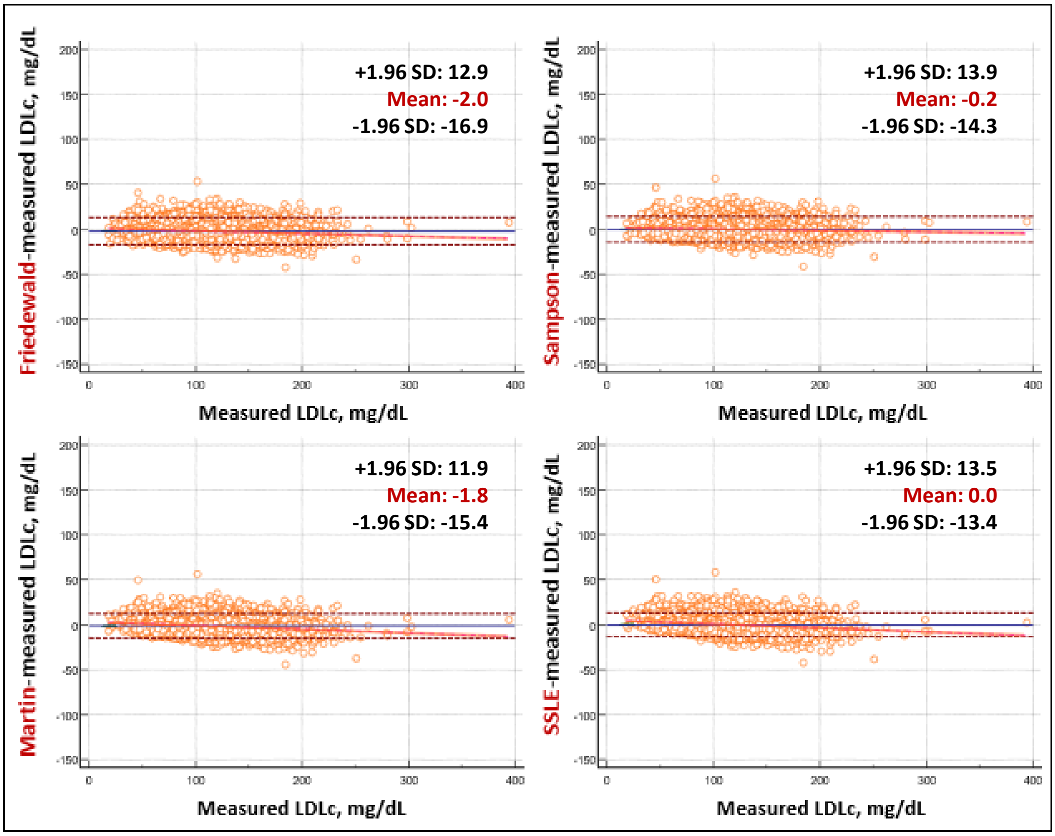

B. TG levels (≥200 to <400 mg/dL), (n=8543)

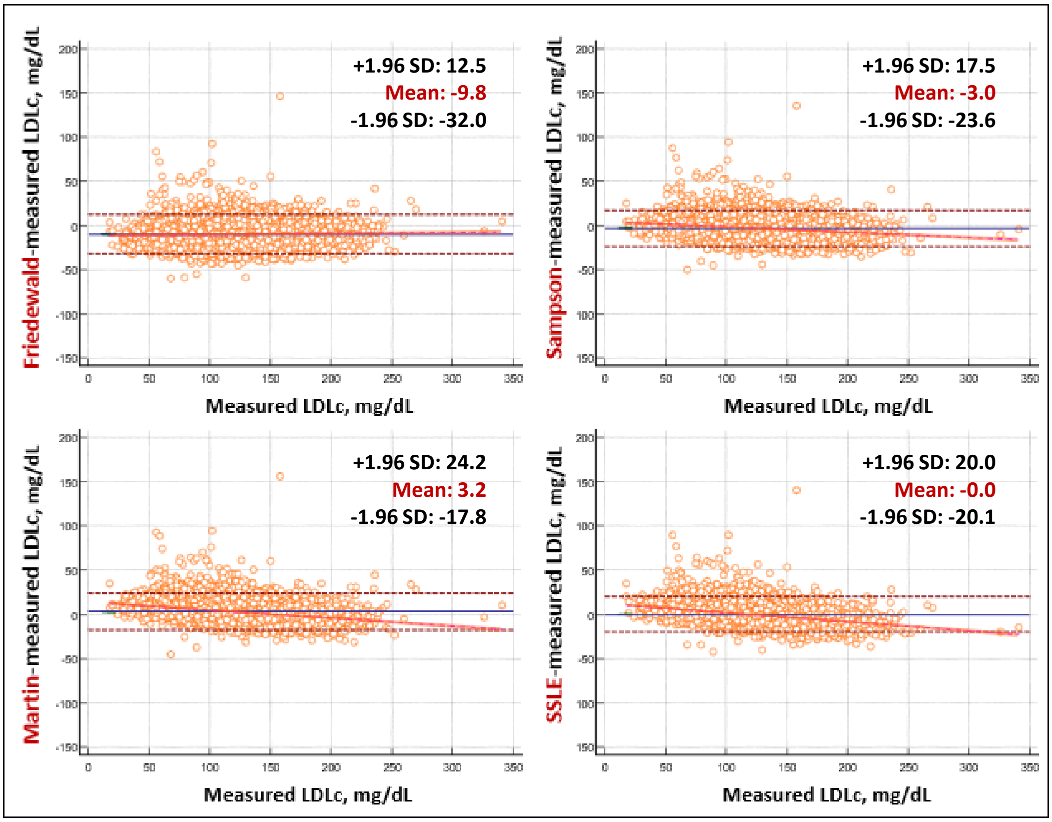

C. TG levels (≥400 to <800 mg/dL), (n=1269)

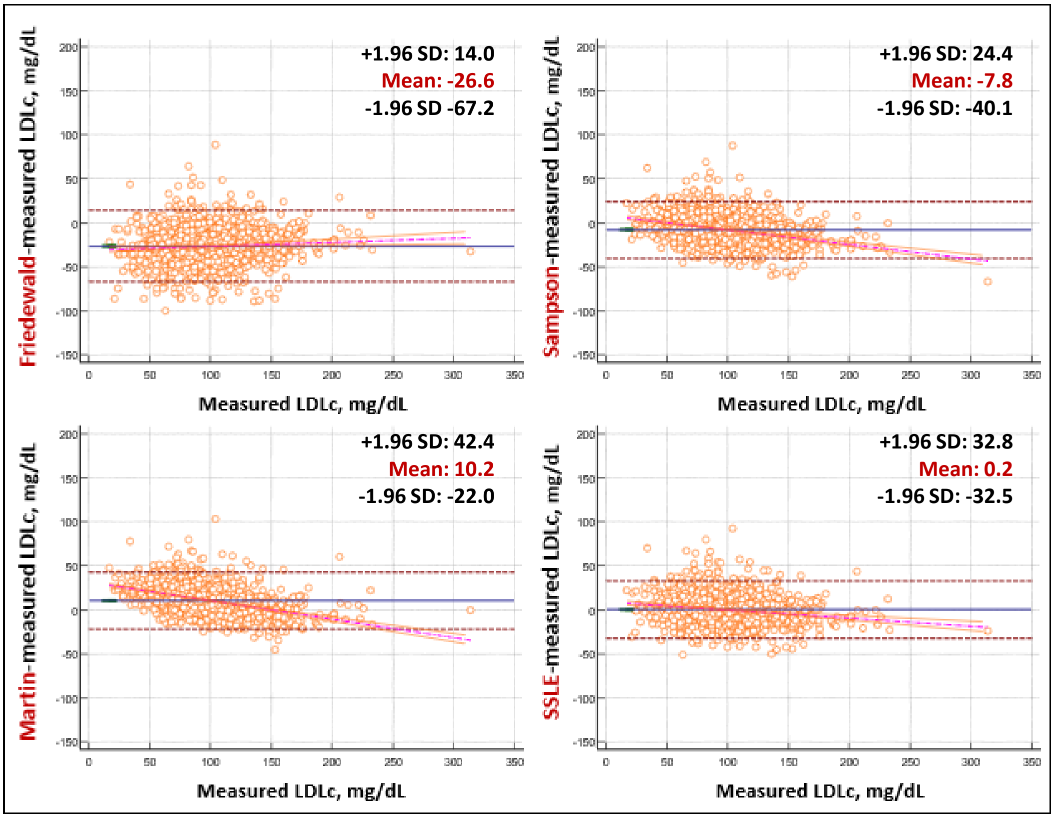

Supplement: Supplementary file 1 [file metabolites-15-00018-s001.zip › Figure S3.pdf]
