# Supplementary material for: The Validation of a Novel, Sex-Specific LDL-Cholesterol Equation and the Friedewald, Sampson-NIH, and Extended-Martin–Hopkins Equations Against Direct Measurement in Korean Adults
Source: Metabolites. 2025 Jan 5;15(1):18. doi: 10.3390/metabo15010018 (PMC11767536; doi:10.3390/metabo15010018)

S4. Bland-Altman Plots Stratified by Low-Density Lipoprotein cholesterol (LDLc) Subgroups

A. LDLc levels <70 mg/dL (n=1957)

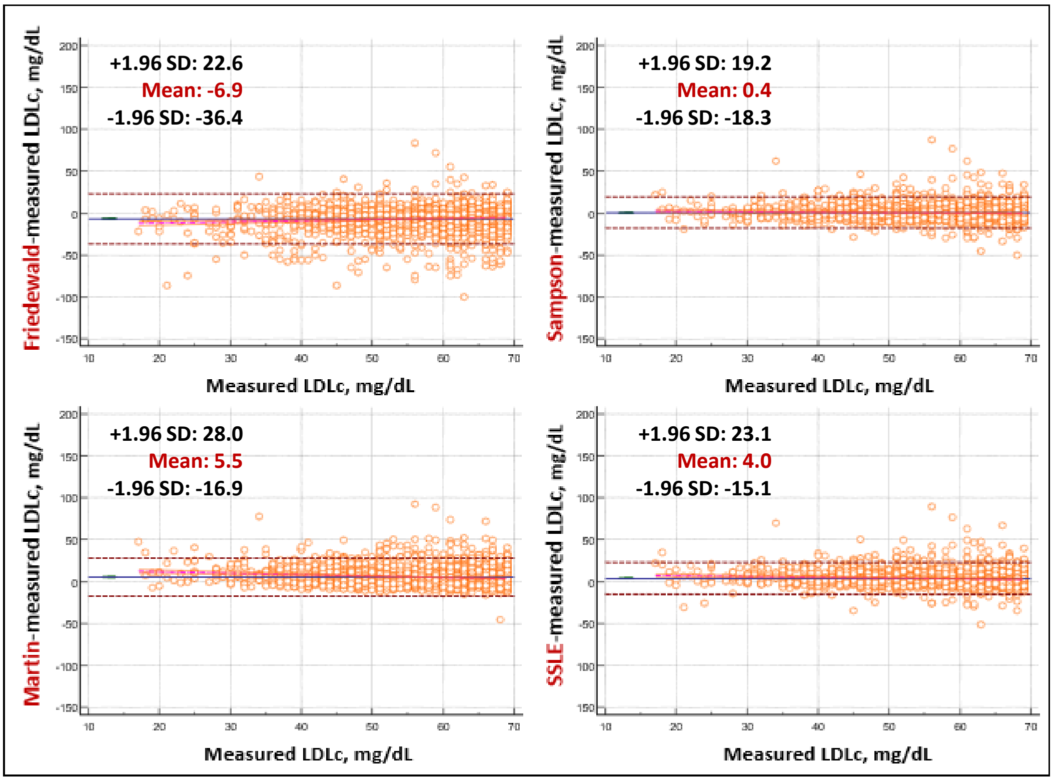

B. LDLc levels ≥70 mg/dL (n=21,800)

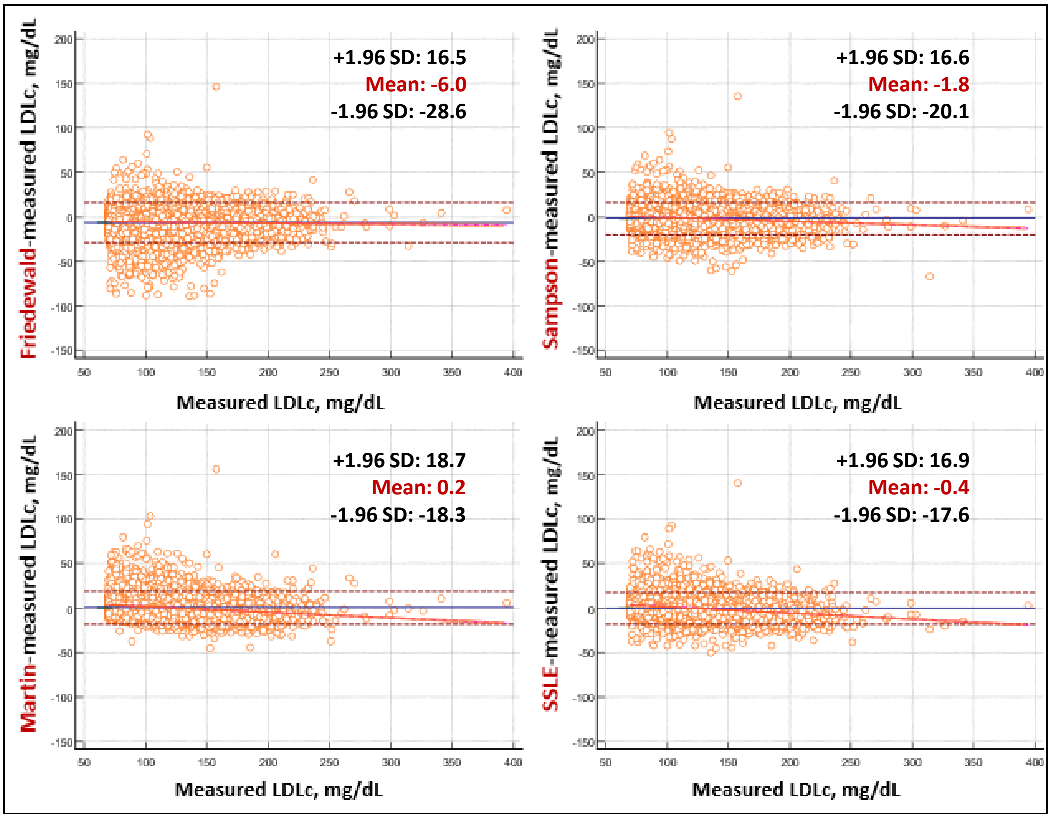

Supplement: Supplementary file 1 [file metabolites-15-00018-s001.zip › Figure S4.pdf]
