# Supplementary material for: The Validation of a Novel, Sex-Specific LDL-Cholesterol Equation and the Friedewald, Sampson-NIH, and Extended-Martin–Hopkins Equations Against Direct Measurement in Korean Adults
Source: Metabolites. 2025 Jan 5;15(1):18. doi: 10.3390/metabo15010018 (PMC11767536; doi:10.3390/metabo15010018)
Supplement: Supplementary file 1 [file metabolites-15-00018-s001.zip › Figure S5.pdf]

S5. Subgroup Analysis by Age, Hypertension, and BMI

Comparison of multiple methods with reference to directly measured LDL cholesterol

[1] Age

- **Group 1 (19-39 years)**
- **Group 2 (40-59 years)**
- Group 3 (60-79 years)
- Group 4 (≥ 80 years)

Reference method	LDLd
Filter	Group 1

Systematic differences

Variable	n	Differences (Variable - Reference)		
		Mean	SD	95% CI
Friedewald	6556	-6.6925	11.8872	-6.9803 to -6.4047
Sampson_2020	6556	-3.1858	8.8405	-3.3998 to -2.9717
Martin_2021	6556	-1.9696	7.9935	-2.1632 to -1.7761
**SSLE**_2024	6556	-1.8540	8.0251	-2.0483 to -1.6597

Limits of agreement

Variable	n	Limits of agreement			
		Lower limit	95% CI	Upper limit	95% CI
Friedewald	6556	-29.9913	-30.4832 to -29.4994	16.6064	16.1145 to 17.0982
Sampson_2020	6556	-20.5131	-20.8789 to -20.1473	14.1416	13.7758 to 14.5074
Martin_2021	6556	-17.6369	-17.9677 to -17.3062	13.6977	13.3669 to 14.0284
**SSLE**_2024	6556	-17.5832	-17.9153 to -17.2511	13.8752	13.5431 to 14.2073

Regression

Variable	Intercept	95% CI	Slope	95% CI	P
Friedewald	0.9410	-0.1064 to 1.9884	-0.06771	-0.07665 to -0.05876	<0.0001
Sampson_2020	4.5054	3.7375 to 5.2734	-0.06822	-0.07478 to -0.06166	<0.0001
Martin_2021	5.5145	4.8236 to 6.2054	-0.06638	-0.07228 to -0.06048	<0.0001
**SSLE**_2024	7.2316	6.5500 to 7.9132	-0.08059	-0.08641 to -0.07477	<0.0001

Absolute percentage error

Variable	n	Median	95% CI	95th Percentile	95% CI
Friedewald	6556	6.37%	6.19 to 6.60	25.90%	24.62 to 27.17
Sampson_2020	6556	5.16%	5.02 to 5.30	16.87%	16.17 to 17.62
Martin_2021	6556	4.59%	4.46 to 4.69	14.57%	13.98 to 14.94
**SSLE**_2024	6556	4.43%	4.31 to 4.53	14.67%	14.04 to 15.42

Reference method	LDLd
Filter	Group 2

Systematic differences

Variable	n	Differences (Variable - Reference)		
		Mean	SD	95% CI
Friedewald	9565	-6.8510	12.5711	-7.1030 to -6.5991
Sampson_2020	9565	-1.7434	9.7957	-1.9398 to -1.5471
Martin_2021	9565	1.1718	10.0196	0.9709 to 1.3726
**SSLE**_2024	9565	0.1408	9.2999	-0.04556 to 0.3272

Limits of agreement

Variable	n	Limits of agreement			
		Lower limit	95% CI	Upper limit	95% CI
Friedewald	9565	-31.4904	-31.9210 to -31.0598	17.7884	17.3577 to 18.2190
Sampson_2020	9565	-20.9430	-21.2786 to -20.6075	17.4561	17.1206 to 17.7917
Martin_2021	9565	-18.4667	-18.8100 to -18.1235	20.8103	20.4670 to 21.1535
**SSLE**_2024	9565	-18.0869	-18.4055 to -17.7683	18.3686	18.0500 to 18.6872

Regression

Variable	Intercept	95% CI	Slope	95% CI	P
Friedewald	-9.0709	-9.9939 to -8.1479	0.01853	0.01112 to 0.02594	<0.0001
Sampson_2020	2.8180	2.1042 to 3.5318	-0.03807	-0.04380 to -0.03234	<0.0001
Martin_2021	11.2189	10.5127 to 11.9251	-0.08386	-0.08953 to -0.07819	<0.0001
**SSLE**_2024	8.8798	8.2208 to 9.5388	-0.07294	-0.07823 to -0.06765	<0.0001

Absolute percentage error

Variable	n	Median	95% CI	95th Percentile	95% CI
Friedewald	9565	6.70%	6.54 to 6.87	29.61%	28.17 to 31.00
Sampson_2020	9565	5.06%	4.95 to 5.18	17.76%	17.29 to 18.42
Martin_2021	9565	4.77%	4.65 to 4.90	19.76%	19.01 to 21.14
**SSLE**_2024	9565	4.61%	4.51 to 4.73	17.32%	16.68 to 17.97

Comparison of multiple methods with reference to directly measured LDL cholesterol

[1] Age

- Group 1 (19-39 years)
- Group 2 (40-59 years)
- **Group 3 (60-79 years)**
- **Group 4 (≥ 80 years)**

Reference method	LDLd
Filter	Group 3

Systematic differences

Variable	n	Differences (Variable - Reference)		
		Mean	SD	95% CI
Friedewald	6972	-4.7910	10.8262	-5.0451 to -4.5368
Sampson_2020	6972	-0.1090	9.1667	-0.3242 to 0.1062
Martin_2021	6972	2.3260	10.2817	2.0847 to 2.5674
**SSLE** _2024	6972	1.4482	9.0115	1.2367 to 1.6598

Limits of agreement

Variable	n	Limits of agreement			
		Lower limit	95% CI	Upper limit	95% CI
Friedewald	6972	-26.0103	-26.4447 to -25.5759	16.4284	15.9940 to 16.8628
Sampson_2020	6972	-18.0758	-18.4437 to -17.7080	17.8578	17.4900 to 18.2256
Martin_2021	6972	-17.8260	-18.2386 to -17.4134	22.4781	22.0655 to 22.8906
**SSLE** _2024	6972	-16.2143	-16.5759 to -15.8527	19.1107	18.7491 to 19.4723

Regression

Variable	Intercept	95% CI	Slope	95% CI	P
Friedewald	-6.9557	-7.7813 to -6.1302	0.01957	0.01247 to 0.02668	<0.0001
Sampson_2020	1.9815	1.2829 to 2.6801	-0.01890	-0.02491 to -0.01289	<0.0001
Martin_2021	9.1406	8.3731 to 9.9080	-0.06161	-0.06822 to -0.05501	<0.0001
**SSLE** _2024	7.8668	7.1966 to 8.5370	-0.05803	-0.06380 to -0.05227	<0.0001

Absolute percentage error

Variable	n	Median	95% CI	95th Percentile	95% CI
Friedewald	6972	6.57%	6.38 to 6.74	26.12%	24.93 to 27.31
Sampson_2020	6972	5.18%	5.05 to 5.35	18.04%	17.38 to 18.61
Martin_2021	6972	5.31%	5.18 to 5.47	23.80%	22.70 to 25.51
**SSLE** _2024	6972	4.71%	4.60 to 4.82	19.16%	18.13 to 20.28

Reference method	LDLd
Filter	Group 4

Systematic differences

Variable	n	Differences (Variable - Reference)		
		Mean	SD	95% CI
Friedewald	664	-3.5345	8.9047	-4.2131 to -2.8560
Sampson_2020	664	0.2214	8.4635	-0.4235 to 0.8663
Martin_2021	664	1.9545	10.3085	1.1690 to 2.7400
**SSLE** _2024	664	1.2510	8.5107	0.6025 to 1.8995

Limits of agreement

Variable	n	Limits of agreement			
		Lower limit	95% CI	Upper limit	95% CI
Friedewald	664	-20.9877	-22.1479 to -19.8275	13.9187	12.7584 to 15.0789
Sampson_2020	664	-16.3671	-17.4699 to -15.2644	16.8099	15.7072 to 17.9126
Martin_2021	664	-18.2501	-19.5933 to -16.9070	22.1591	20.8160 to 23.5022
**SSLE** _2024	664	-15.4299	-16.5388 to -14.3210	17.9319	16.8230 to 19.0408

Regression

Variable	Intercept	95% CI	Slope	95% CI	P
Friedewald	-4.1650	-6.2616 to -2.0684	0.005886	-0.01263 to 0.02440	0.5328
Sampson_2020	1.0208	-0.9715 to 3.0131	-0.007462	-0.02506 to 0.01013	0.4053
Martin_2021	5.0739	2.6591 to 7.4887	-0.02912	-0.05045 to -0.007792	0.0075
**SSLE** _2024	5.5445	3.5701 to 7.5188	-0.04008	-0.05752 to -0.02264	<0.0001

Absolute percentage error

Variable	n	Median	95% CI	95th Percentile	95% CI
Friedewald	664	6.79%	6.19 to 7.47	20.83%	17.85 to 24.22
Sampson_2020	664	5.37%	4.99 to 5.80	16.98%	15.34 to 19.86
Martin_2021	664	6.13%	5.59 to 6.58	23.86%	20.65 to 26.85
**SSLE** _2024	664	4.90%	4.45 to 5.25	18.59%	15.97 to 23.01

Comparison of multiple methods with reference to directly measured LDL cholesterol

[2] Hypertension

Reference method	LDLd

Filter	HTN=1<br>Hypertension

Systematic differences

Variable	n	Differences (Variable - Reference)		
		Mean	SD	95% CI
Friedewald	9032	-7.4572	12.0852	-7.7064 to -7.2079
Sampson_2020	9032	-2.0090	9.5881	-2.2068 to -1.8113
Martin_2021	9032	1.3291	10.3431	1.1158 to 1.5424
**SSLE** _2024	9032	0.2779	9.2775	0.08653 to 0.4692

Limits of agreement

Variable	n	Limits of agreement			
		Lower limit	95% CI	Upper limit	95% CI
Friedewald	9032	-31.1442	-31.5702 to -30.7182	16.2299	15.8039 to 16.6559
Sampson_2020	9032	-20.8017	-21.1397 to -20.4637	16.7837	16.4457 to 17.1217
Martin_2021	9032	-18.9433	-19.3079 to -18.5787	21.6015	21.2369 to 21.9661
**SSLE** _2024	9032	-17.9061	-18.2331 to -17.5790	18.4618	18.1348 to 18.7889

Regression

Variable	Intercept	95% CI	Slope	95% CI	P
Friedewald	-9.3069	-10.1237 to -8.4901	0.01651	0.009569 to 0.02346	<0.0001
Sampson_2020	1.5153	0.8710 to 2.1596	-0.03146	-0.03694 to -0.02599	<0.0001
Martin_2021	9.6762	9.0001 to 10.3524	-0.07452	-0.08027 to -0.06877	<0.0001
**SSLE** _2024	7.6509	7.0437 to 8.2580	-0.06582	-0.07098 to -0.06066	<0.0001

Absolute percentage error

Variable	n	Median	95% CI	95th Percentile	95% CI
Friedewald	9032	7.62%	7.47 to 7.80	30.93%	29.77 to 32.25
Sampson_2020	9032	5.53%	5.41 to 5.66	18.68%	18.16 to 19.44
Martin_2021	9032	5.41%	5.28 to 5.53	23.14%	21.90 to 24.25
**SSLE** _2024	9032	4.85%	4.74 to 4.96	18.70%	17.98 to 19.83

Comparison of multiple methods with reference to directly measured LDL cholesterol

[3] Body Mass Index (BMI)

- **Group 1 (< 18.5 kg/m^2)**
- **Group 2 (18.5 – 22.9 kg/m^2)**
- Group 3 (23.0 – 24.9 kg/m^2)
- Group 4 (≥ 25.0 kg/m^2)-> Obesity in Asian

Reference method	LDLd
Filter	Group 1

Systematic differences

Variable	n	Differences (Variable - Reference)		
		Mean	SD	95% CI
Friedewald	704	-1.4582	8.9497	-2.1205 to -0.7960
Sampson_2020	704	0.3230	7.6848	-0.2456 to 0.8917
Martin_2021	704	-0.8823	7.7167	-1.4533 to -0.3113
SSLE _2024	704	0.8206	7.2820	0.2817 to 1.3594

Limits of agreement

Variable	n	Limits of agreement			
		Lower limit	95% CI	Upper limit	95% CI
Friedewald	704	-18.9996	-20.1319 to -17.8673	16.0831	14.9508 to 17.2155
Sampson_2020	704	-14.7393	-15.7116 to -13.7670	15.3853	14.4130 to 16.3576
Martin_2021	704	-16.0069	-16.9833 to -15.0306	14.2423	13.2660 to 15.2187
SSLE _2024	704	-13.4522	-14.3735 to -12.5309	15.0934	14.1720 to 16.0147

Regression

Variable	Intercept	95% CI	Slope	95% CI	P
Friedewald	2.6289	0.2053 to 5.0525	-0.04137	-0.06498 to -0.01776	0.0006
Sampson_2020	3.3968	1.3117 to 5.4820	-0.03111	-0.05142 to -0.01080	0.0027
Martin_2021	5.6071	3.5599 to 7.6542	-0.06568	-0.08562 to -0.04574	<0.0001
SSLE _2024	8.1982	6.2925 to 10.1038	-0.07467	-0.09323 to -0.05610	<0.0001

Absolute percentage error

Variable	n	Median	95% CI	95th Percentile	95% CI
Friedewald	704	6.07%	5.68 to 6.49	18.26%	15.94 to 21.66
Sampson_2020	704	5.42%	5.05 to 5.83	16.21%	15.25 to 18.65
Martin_2021	704	5.39%	4.94 to 5.76	15.83%	14.64 to 19.51
SSLE _2024	704	5.07%	4.60 to 5.64	15.93%	14.21 to 19.47

Reference method	LDLd
Filter	Group 2

Systematic differences

Variable	n	Differences (Variable - Reference)		
		Mean	SD	95% CI
Friedewald	11256	-3.6596	11.0466	-3.8637 to -3.4555
Sampson_2020	11256	-0.09695	8.8645	-0.2607 to 0.06683
Martin_2021	11256	0.7853	9.1217	0.6168 to 0.9539
SSLE _2024	11256	0.9043	8.4496	0.7482 to 1.0604

Limits of agreement

Variable	n	Limits of agreement			
		Lower limit	95% CI	Upper limit	95% CI
Friedewald	11256	-25.3109	-25.6597 to -24.9620	17.9916	17.6428 to 18.3405
Sampson_2020	11256	-17.4713	-17.7512 to -17.1914	17.2774	16.9975 to 17.5573
Martin_2021	11256	-17.0932	-17.3812 to -16.8051	18.6638	18.3758 to 18.9519
SSLE _2024	11256	-15.6569	-15.9237 to -15.3900	17.4655	17.1987 to 17.7323

Regression

Variable	Intercept	95% CI	Slope	95% CI	P
Friedewald	-2.8167	-3.5448 to -2.0886	-0.007521	-0.01376 to -0.001285	0.0181
Sampson_2020	2.9398	2.3584 to 3.5213	-0.02710	-0.03208 to -0.02212	<0.0001
Martin_2021	7.4770	6.8896 to 8.0644	-0.05971	-0.06474 to -0.05468	<0.0001
SSLE _2024	7.6786	7.1371 to 8.2202	-0.06045	-0.06509 to -0.05581	<0.0001

Absolute percentage error

Variable	n	Median	95% CI	95th Percentile	95% CI
Friedewald	11256	5.62%	5.50 to 5.76	23.78%	22.86 to 24.77
Sampson_2020	11256	4.84%	4.73 to 4.94	16.58%	16.09 to 17.27
Martin_2021	11256	4.62%	4.53 to 4.72	18.05%	17.35 to 18.87
SSLE _2024	11256	4.43%	4.33 to 4.52	16.76%	16.33 to 17.48

Comparison of multiple methods with reference to directly measured LDL cholesterol

[3] Body Mass Index (BMI)

- Group 1 (< 18.5 kg/m^2)
- Group 2 (18.5 – 22.9 kg/m^2)
- **Group 3 (23.0 – 24.9 kg/m^2)**
- **Group 4 (≥ 25.0 kg/m^2)-> Obesity in Asian**

Reference method	LDLd
Filter	Group 3

Systematic differences

Variable	n	Differences (Variable - Reference)		
		Mean	SD	95% CI
Friedewald	7606	-7.7764	12.3734	-8.0545 to -7.4982
Sampson_2020	7606	-2.4393	9.7278	-2.6579 to -2.2206
Martin_2021	7606	0.9724	9.9542	0.7487 to 1.1962
**SSLE** _2024	7606	-0.4604	9.2585	-0.6685 to -0.2523

Limits of agreement

Variable	n	Limits of agreement			
		Lower limit	95% CI	Upper limit	95% CI
Friedewald	7606	-32.0281	-32.5035 to -31.5528	16.4754	16.0001 to 16.9508
Sampson_2020	7606	-21.5057	-21.8794 to -21.1320	16.6271	16.2534 to 17.0008
Martin_2021	7606	-18.5379	-18.9203 to -18.1555	20.4827	20.1003 to 20.8651
**SSLE** _2024	7606	-18.6071	-18.9627 to -18.2514	17.6862	17.3306 to 18.0419

Regression

Variable	Intercept	95% CI	Slope	95% CI	P
Friedewald	-10.2802	-11.2750 to -9.2854	0.02105	0.01302 to 0.02908	<0.0001
Sampson_2020	1.6456	0.8680 to 2.4232	-0.03434	-0.04062 to -0.02806	<0.0001
Martin_2021	10.1635	9.3912 to 10.9358	-0.07727	-0.08351 to -0.07104	<0.0001
**SSLE** _2024	7.4812	6.7591 to 8.2033	-0.06677	-0.07260 to -0.06094	<0.0001

Absolute percentage error

Variable	n	Median	95% CI	95th Percentile	95% CI
Friedewald	7606	7.05%	6.81 to 7.23	30.23%	28.75 to 31.45
Sampson_2020	7606	5.16%	5.03 to 5.29	18.17%	17.45 to 18.79
Martin_2021	7606	4.72%	4.60 to 4.85	20.10%	18.93 to 21.23
**SSLE** _2024	7606	4.52%	4.41 to 4.64	17.30%	16.45 to 17.98

Reference method	LDLd
Filter	Group 4

Systematic differences

Variable	n	Differences (Variable - Reference)		
		Mean	SD	95% CI
Friedewald	4033	-10.5547	11.5537	-10.9114 to -10.1980
Sampson_2020	4033	-4.5058	9.6286	-4.8030 to -4.2085
Martin_2021	4033	0.1119	11.1926	-0.2336 to 0.4574
**SSLE** _2024	4033	-1.6881	9.6576	-1.9863 to -1.3900

Limits of agreement

Variable	n	Limits of agreement			
		Lower limit	95% CI	Upper limit	95% CI
Friedewald	4033	-33.2001	-33.8097 to -32.5904	12.0906	11.4809 to 12.7002
Sampson_2020	4033	-23.3778	-23.8858 to -22.8697	14.3662	13.8581 to 14.8743
Martin_2021	4033	-21.8256	-22.4162 to -21.2350	22.0494	21.4588 to 22.6400
**SSLE** _2024	4033	-20.6170	-21.1266 to -20.1074	17.2407	16.7311 to 17.7503

Regression

Variable	Intercept	95% CI	Slope	95% CI	P
Friedewald	-10.8088	-12.0026 to -9.6150	0.002160	-0.007525 to 0.01185	0.6619
Sampson_2020	1.8298	0.8562 to 2.8033	-0.05386	-0.06176 to -0.04596	<0.0001
Martin_2021	10.5204	9.4140 to 11.6267	-0.08848	-0.09746 to -0.07951	<0.0001
**SSLE** _2024	7.9244	6.9763 to 8.8726	-0.08172	-0.08941 to -0.07402	<0.0001

Absolute percentage error

Variable	n	Median	95% CI	95th Percentile	95% CI
Friedewald	4033	8.73%	8.46 to 8.99	32.05%	29.79 to 33.90
Sampson_2020	4033	5.90%	5.70 to 6.11	19.05%	18.24 to 19.93
Martin_2021	4033	5.87%	5.67 to 6.02	23.34%	21.82 to 24.80
**SSLE** _2024	4033	5.07%	4.95 to 5.21	17.95%	17.01 to 19.67
