# Supplementary figures and images for: The Validation of a Novel, Sex-Specific LDL-Cholesterol Equation and the Friedewald, Sampson-NIH, and Extended-Martin–Hopkins Equations Against Direct Measurement in Korean Adults
Source: Metabolites. 2025 Jan 5;15(1):18. doi: 10.3390/metabo15010018 (PMC11767536; doi:10.3390/metabo15010018)

## S2. Bland-Altman Plots Stratified by Sex

### A. Males (n=12,094)

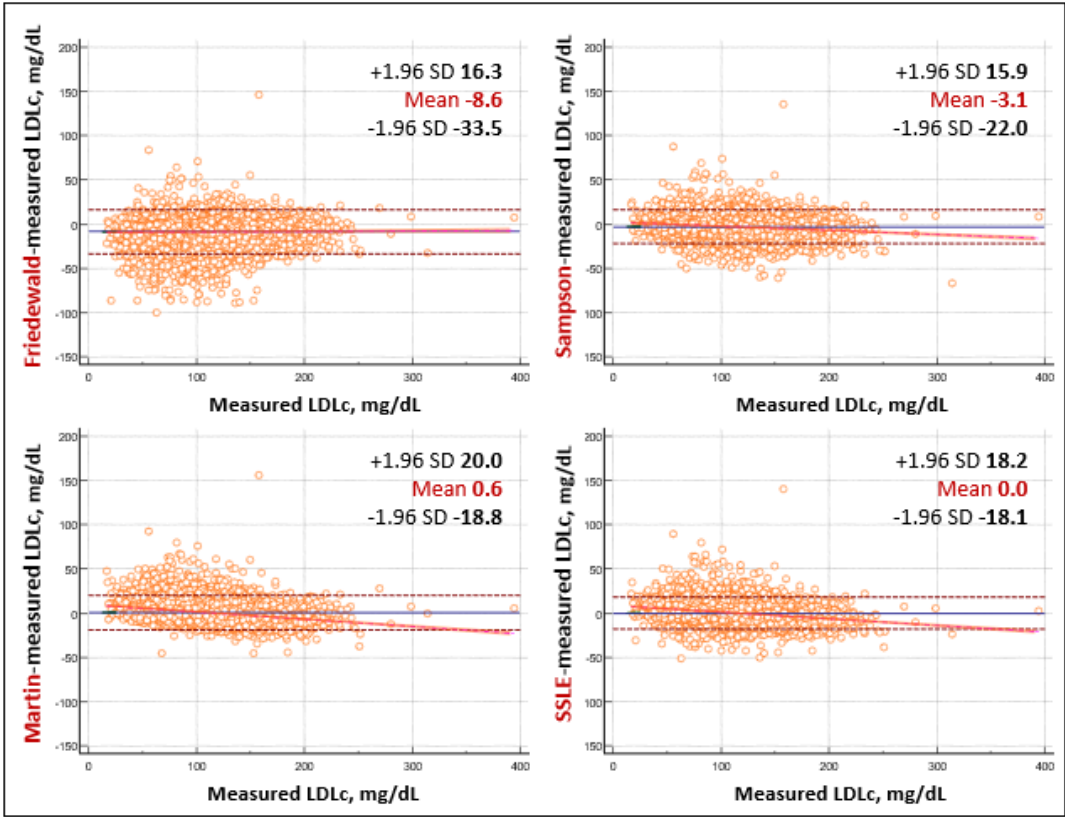

### B. Females (n=11,663)

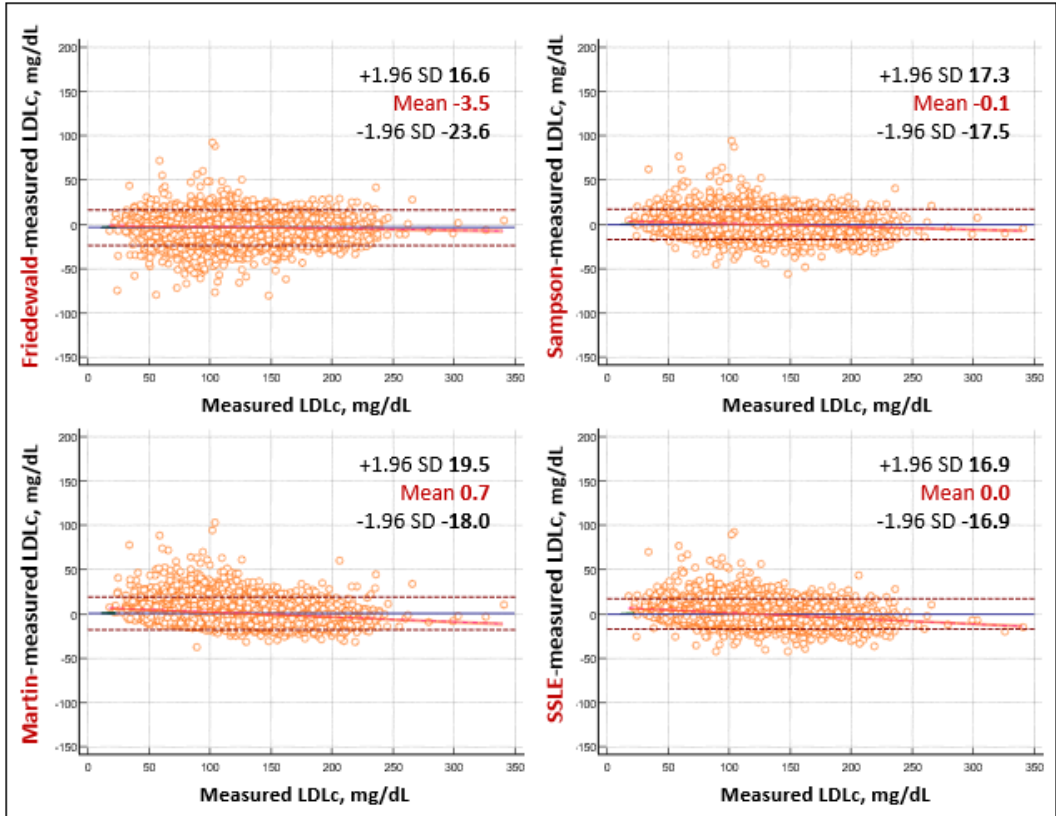

Supplement: Supplementary file 1 [file metabolites-15-00018-s001.zip › Figure S2.pdf]
